# A TEST OF THE OKLAHOMA INMATE FORGIVENESS AND WELL-BEING MODEL

**DOI:** 10.1093/geroni/igz038.1305

**Published:** 2019-11-08

**Authors:** George K Randall, Alex Bishop

**Affiliations:** 1 Sam Houston State University, Huntsville, Texas, United States; 2 Oklahoma State University, Stillwater, Oklahoma, United States

## Abstract

Data was collected from older male offenders (N = 86 non-violent; N = 163 violent) incarcerated in Oklahoma. Testing a forgiveness model, positive evaluation of life PVOL was regressed on religiosity (REL) and forgiveness of self (FSelf), others (FOthers), and situation (FSit) using hierarchical OLS regression. Blocks of predictors included: a) age and education; b) religiosity; and c) FSelf, FOthers, and FSit. For the non-violent model of PVOL significant predictors included REL (β = .26, p ≤ .01) and FSelf (β = .40, p ≤ .01). For the violent offender model of PVOL significant predictors included REL (β = .31, p ≤ .001), FS (β = .21, p ≤ .01) and FSit (β = .33, p ≤ .001). Result indicate effects of REL and FSelf for both non-violent and violent offenders but a unique association of FSit for violent offenders. Implications for gerontological inquiry, practice, and policy are discussed.

